# Modelling the GDP of KSA using linear and non-linear NNAR and hybrid stochastic time series models

**DOI:** 10.1371/journal.pone.0297180

**Published:** 2024-02-23

**Authors:** Abdullah M. Almarashi, Muhammad Daniyal, Farrukh Jamal

**Affiliations:** 1 Department of Statistics, Faculty of Science, King Abdulaziz University, Jeddah, Saudi Arabia; 2 Department of Statistics, The Islamia University of Bahawalpur, Punjab, Pakistan; Cairo University, EGYPT

## Abstract

**Background:**

Gross domestic product (GDP) serves as a crucial economic indicator for measuring a country’s economic growth, exhibiting both linear and non-linear trends. This study aims to analyze and propose an efficient and accurate time series approach for modeling and forecasting the GDP annual growth rate (%) of Saudi Arabia, a key financial indicator of the country.

**Methodology:**

Stochastic linear and non-linear time series modeling, along with hybrid approaches, are employed and their results are compared. Initially, conventional linear and nonlinear methods such as ARIMA, Exponential smoothing, TBATS, and NNAR are applied. Subsequently, hybrid models combining these individual time series approaches are utilized. Model diagnostics, including mean absolute error (MAE), root mean square error (RMSE), and mean absolute percentage error (MAPE), are employed as criteria for model selection to identify the best-performing model.

**Results:**

The findings demonstrated that the neural network autoregressive (NNAR) model, as a non-linear approach, outperformed all other models, exhibiting the lowest values of MAE, RMSE and MAPE. The NNAR(5,3) projected the GDP of 1.3% which is close to the projection of IMF benchmark (1.9) for the year 2023.

**Conclusion:**

The selected model can be employed by economists and policymakers to formulate appropriate policies and plans. This quantitative study provides policymakers with a basis for monitoring fluctuations in GDP growth from 2022 to 2029 and ensuring the sustained progression of GDP beyond 2029. Additionally, this study serves as a guide for researchers to test these approaches in different economic dynamics.

## 1. Introduction

The real Gross Domestic Product (GDP) serves as a comprehensive measure of economic activity, encompassing the total value of goods and services produced within an economy. This metric holds significance across academic, investment, and regulatory circles, serving as a representation of economic wealth and a crucial informative gauge that steers decision-making processes [1]. Indeed, there is a notable interest in directing attention towards not only national economic policies but also other domains, ranging from addressing non-performing loans [2] to addressing the natural disasters [3]. Several methodologies have been added in existing literature to predict the Gross Domestic Product (GDP). Notably, the macroeconomic discourse investigating this subject through a time series approach employed various specifications of Vector Autoregression (VAR) models [4–6]. A study conducted by [7] introduced a comparison encompassing reduced form, autoregressive, VAR, and Markov switching models, and it was found that a simple autoregressive process of second order [AR (2)] within the time series framework yields superior performance in forecasting the GDP of the United States. An additional investigation conducted by [8] presented an illustration of employing ARIMA for predicting the GDP of the European Union. As indicated by [9], the act of forecasting, in a broader sense, encompasses diverse techniques and models, including dynamic regression models, ARIMA models, and advanced forecasting approaches like the Neural Network Model [9]. Accorinding to it, various studies analyzing GDP growth have employed diverse categories of prediction models, including linear and non-linear regression models, time series models, and models based on artificial neural networks, all aimed at predicting GDP growth. Among the time series models are the Autoregressive model (AR), the Moving Average model (MA), and the Autoregressive Integrated Moving Average model (ARIMA). Additional statistical models employed in forecasting comprise non-linear multiple regression models like Bilinear models, GARCHand the Threshold Autoregressive (TAR) models.

The methods mentioned earlier are the main techniques used in the field of economic prediction. These methods use linear models to simulate the real-world problems for prediction purposes. However, they don’t always yield accurate outcomes when dealing with non-linear series [10]. Recognizing the shortcomings of these linear models, many researchers have shifted their focus toward non-linear models. Initially, as researchers began exploring non-linear models, they stuck to the conventional methods of modeling [11–13]. In the practical forecasting procedure, these methods experience information loss owing to challenges like multicollinearity and error series, resulting in unsatisfactory predictive accuracy. In reality, GDP is subject to the influence of multiple factors with intricate interrelationships, leading to complex time series and non-linearity. This complexity significantly complicates the task of GDP forecasting. In the face of unsolvable complex non-linear problems, people have started to look beyond traditional methods to find new ways of research. The human brain can handle a variety of complex non-linear problems very quickly, and researchers have been inspired by the idea of how to simulate the human brain to deal with complex non-linear problems. An artificial neural network (ANN) is an intelligent based model that make use of the function of neurons in the brain [14–17], and is a non-linear complex network system consisting of a large number of interconnected neurons.

Currently, research on neural network-based forecasting has focused on time series forecasting Doucoure et al. [18] showed that ANN has higher performance than ARMA. Prior studies have demonstrated that ANN holds the potential to deliver remarkable outcomes in tasks involving the prediction of time series data [19–23]. Tkacz [24] similarly employed neural networks to forecast the Canada’s actual GDP which concluded that neural networks yield more effective year-to-year predictions of real GDP growth as compared to other alternative models. Numerous studies suggest employing hybrid models due to the challenge of distinguishing linear from non-linear processes in practical analysis of time series data [25]. Further, according to [25], it is very rare that real time series have the purely linear or non-linear pattern and it may be possible that both linear and non-linear patterns exist. The study explored that by combining different methods, the problem of model selection can be eased. Various studies show that when different models are combined, forecasts are often better compared to using just one model. Different studies proposed different ways to combine models for predicting time series data, and this leads to creating hybrid models. One example of these hybrid models includes using both the linear ARIMA model and the non-linear neural network model (NN) together. This helps capture different types of relationships in the time series information [26]. Different studies have outlined these hybrid models as developed and used over time such as [26, 27]. Various investigations have also addressed economic matters pertaining to Saudi Arabia [28]. The dynamic nonlinear neural network autoregressive (NNAR) models represent neural network architectures that prove valuable in handling such scenarios [29, 30].

The GDP of KSA experienced significant fluctuations between 1969 and 2021, making it challenging for conventional methods like ARIMA to capture hidden non-linear patterns. To address this, we employed specific neural network architectures in our study to predict GDP and enhance modeling. By comparing linear and non-linear techniques, we analyzed annual GDP growth (%) and immediate forecasts. Our assessment included hybrid models (ARIMA, ETS, NNAR, TBATS) for both linear and non-linear time series. These findings can aid the government and stakeholders in devising effective economic policies for improving KSA’s economic indicators.

## 2. Data

The data series used is made up of Saudi Arabia’s GDP growth rate (%) data from 1969 to 2021 from the official website of the World Bank. The descriptive analysis of the data shows that the minimum GDP annual growth of KSA was observed at -20.73% during the year 1982. The average GDP annual growth was observed at 4.830% and the median GDP annual growth was 2.893% with a standard deviation of 11.641. If the average is greater so a large number of years admit GDP values much higher than in the majority of years. The maximum GDP annual growth of KSA was observed at 58.69% during the year 1970. Table 1 shows the descriptive statistics related to the GDP series.

**Table 1:** Descriptive statistics of KSA’s GDP growth (annual %) from 1960 to 2021.

Minimum value	First Quartile	Median value	Mean value	Third Quartile	Maximum value	Standard Deviation
-20.73	-0.9761	2.893	4.830	8.977	58.65	11.641

## 3. Methods

This section deals with the conventional and non-conventional methods used in the study.

### 3.1. Autoregressive integrated moving average (ARIMA)

ARIMA model can be expressed as ARIMA (p, d, q), where, p is AR order, d is thedifferenced trend, and q is the MA order [31]. The AR (p) model identifies that Y_t_ depends upon linearly with its lagged values and on the current residuals ε_t_, and MA (q) shows that Y_t_ depends linearly on the present and its pastresiduals terms (ε_t−1_−ε_t−2_,…,ε_t−q_). AR (p) and MA (q) time series models can be expressed as Eq (1) and Eq (2) consecutively;

$$Y_{t}=\varphi_{1}Y_{t-1}+\varphi_{2}Y_{t-2}+\cdots +\varphi_{p}Y_{t-p}+\varepsilon_{t},$$ (1)

$$Y_{t}=\theta_{1}\varepsilon_{t-1}-\theta_{2}\varepsilon_{t-2}-\cdots -\theta_{q}\varepsilon_{t-q}+\varepsilon_{t},$$ (2)

where Y_t_ is observed or output value of time series, φ and θ are the coefficients of AR and MA models respectively and ε_t_ shows the residual value at time t. Moreover, stochastic terms ε_t_~iid (0, σ^2^). Autoregressive-Moving-Average (ARMA) model is developed by combing the MA and AR models known as ARMA (p, q) models. The ARMA model can be generally represented by the following equation where α shows the constant term, and ε_t−1_ is the past residual noise term.


$$Y_{t}=\alpha +\varphi_{1}Y_{t-1}+\varphi_{2}Y_{t-2}+\cdots +\varphi_{p}Y_{t-p}+\varepsilon_{t}-\theta_{1}\varepsilon_{t-1}-\theta_{2}\varepsilon_{t-2}-\cdots -\theta_{q}\varepsilon_{t-q},$$ (3)

**Fig 1:**
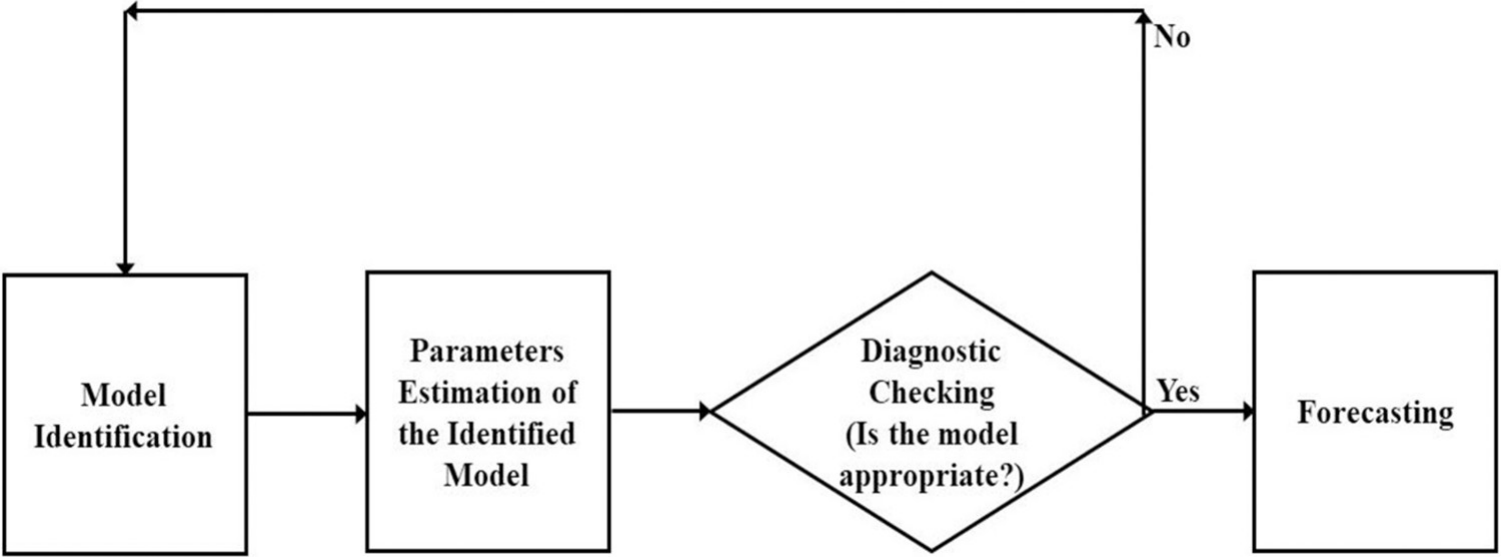
Flowchart of steps involved in the modelling of ARIMA.

The ARIMA modeling methodology consists of four basic iterative steps;

1. **Identification of the ARIMA model** requires that time series data should be stationary. Stationarity implies that the statistical properties of the data, such as mean and variance, remain constant over time. To make the data more agreeable to modeling, the patterns need to be removed. Differencing, as mentioned earlier, is a common technique used to achieve this. It involves subtracting adjacent observations to eliminate trends. The Augmented Dickey-Fuller (ADF) test is a statistical test used to check whether a series is stationary or not.
2. **In the Model estimation,** Once the data series becomes stationary, ACF and PACF are employed. These tools help to understand the relationship between data points at different time lags.
3. **In Diagnostic checks**, the selected candidate model is passed through some tests, which involves creating an ACF plot to assess residual normality, using a QQ-Norm plot, and conducting a Shapiro-Wilk test to examine residual normality. Once the model passes all these tests, the model is then considered one of the best models for the next step.
4. **In the step of Forecasting,** we use the candidate model for forecasting the data series which fulfills the three-step conditions stated early. The flowchart of all four steps explained for modeling the ARIMA model are shown in Fig 1

### 3.2 Non-Linear time series stochastic models

**TBATS** is a non-linear time series model which handles the data series having several seasonal patterns, i.e., the pattern of the data changes its behavior over time. This method is preferred over BATS as Trigonometric seasonality (TBATS) can cope with complicated and high frequencies.

The TBATS model can be expressed as follow;

$$x_{t}^{\left(\alpha \right)}=\tau_{t-1}+\beta k_{t-1}+\sum_{i=1}^{T}\alpha_{t-n_{i}}^{\left(i\right)}+\vartheta_{t}$$

$$\tau_{t}=\tau_{t-1}+\beta k_{t-1}+\gamma \vartheta_{t}$$

$$k_{t}=\beta k_{t-1}+\omega \vartheta_{t}$$

$$\vartheta_{t}=\sum_{i=1}^{p}\delta_{i}\vartheta_{t-1}+\sum_{i=1}^{q}\theta_{i}\varepsilon_{t-1}+\varepsilon_{t}$$ (4)

TBATS models were applied to the data series by using the “tbats()” function.

**Fig 2:**
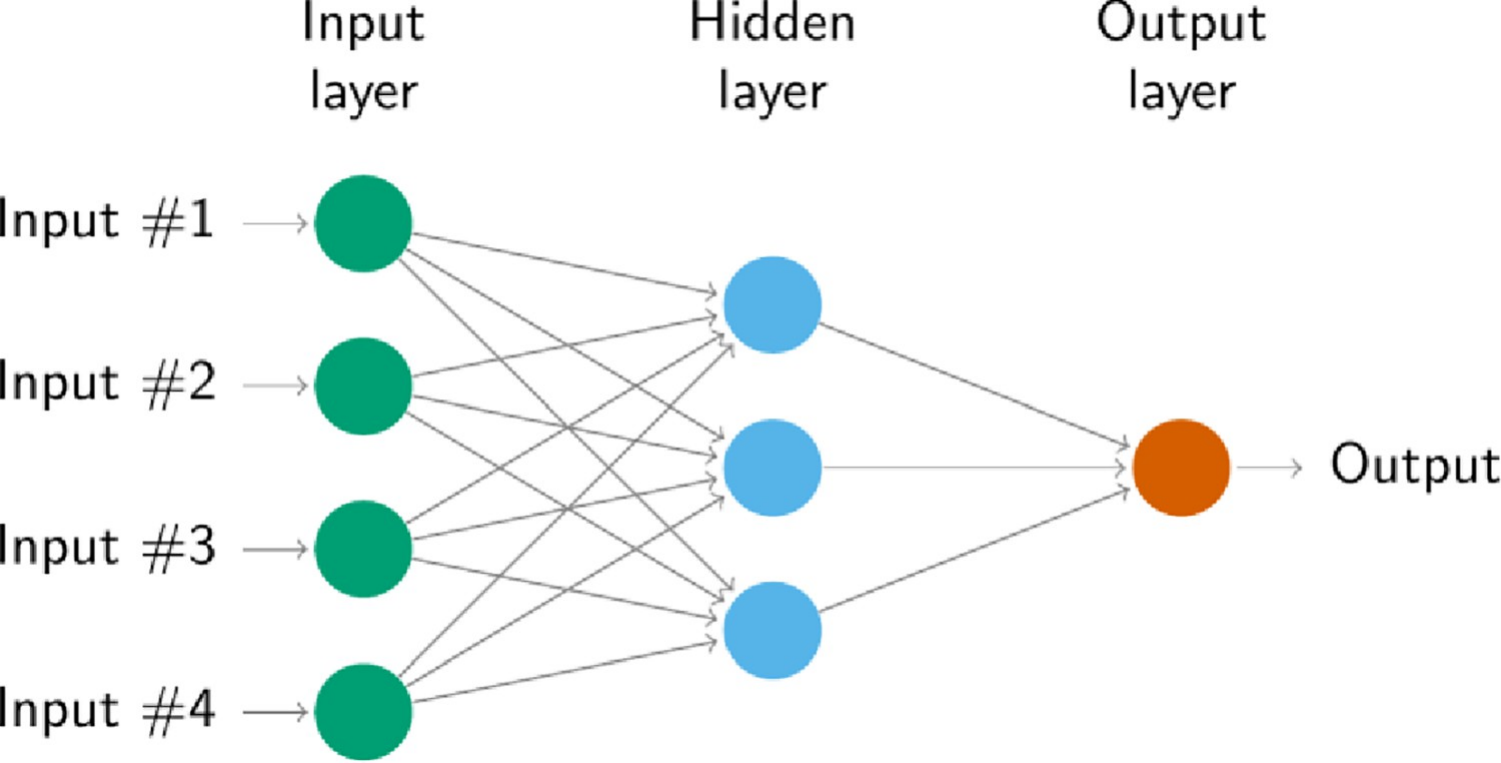
NNAR model with input, hidden, and output layers.

**NNAR** non-linear time series models, type of ANN models, can be considered as a network of nodes or neurons that show complicated non-linear relationships. In the late 1990s, different application areas began to show increased interest in forecasting techniques based on ANN. The application of neural networks in supervised classification, prediction, and nonlinear time series forecasting is widespread. diagrammatical organization of NNAR model with input, hidden, and output layers are shown in Fig 2. In its framework, the neurons are arranged in two layers. The first layer is a bottom layer which shows the original time series and the second top layer shows the prediction or forecasting. An NNAR (*p*, *P*, *k*)_m_ [32, 33] hasthe inputs(y_t−1_, y_t−2_,…,y_t−p_, y_t−m_, y_t−2m_,…y_t−Pm_). TheNNAR is represented by the following form;

$$f(Y)=\beta_{0}+\sum_{k=1}^{K}\beta_{k}g(w_{k0}+\sum_{j=1}^{p}w_{kj}X_{j})$$ (5)

The expression is constructed in two stages. The *K* activations come first. In the activation, *A*(*k*), *k* = 1,…,*K*, the hidden layer is calculated as a function of the the input characteristics *X*_*j*_ = *X*_*t*−1_,…,*X*_*t*−*p*_, with

$$A(k)=h(k)=g\left(w_{k0}+\sum_{j=1}^{p}w_{kj}X_{j}\right)$$ (6)

where *g* is a previously defined nonlinear activation function. Each *A*(*k*) may be seen as a separate *h*_*k*_(*X*) transformation of the unique characteristics. The output layer is presented with these *K* stimulations originating from the hidden layer.


$$f(Y)=\beta_{0}+\sum_{k=1}^{K}\beta_{k}A(k)$$ (7)

For NNAR modeling, a sigmoid activation function is employed, resembling the logistic model. This function transforms a linear function, converting probabilities from 0 to 1. This sigmoid activation has the following functional form;

$$g(z)=\frac{e^{z}}{1+e^{z}}=1/(1+e^{-z})$$ (8)

**ETS** or exponential smoothing can be applied to data having both systematic trends and seasonal components. It is a significant forecasting methodology that can be applied as an alternative to ARIMA techniques. The basic model of the non-linear ETS consists of two equations: (i) a forecast equation and (ii) a smoothing equation. The state space formulation of Holt’s method is:

$$\begin{array}{c} x_{t}=x_{t-1}+e_{t}\\ l_{t}=l_{t-1}+{\alpha e}_{t} \end{array}$$ (9)

This state space formulation can be turned into a procedural formation, a forecast, and a smoothing equation;

$${\hat{x}}_{t-1}=l_{t-1}$$

$$l_{t}=\propto x_{t-1}+(1-\propto )l_{t-1}$$ (10)

Here, ${\hat{x}}_{t-1}$ is the forecast/expectation *x*_*t*_ given the information from the previous step. ETS models were evaluated by using the R-package “ets()” function.

To assess the performance of all forecasting model, we used RMSE, MAE and MAPE which can be explained as follow.

MAE is the average of all absolute errors. The formula is shown below;

$$MAE=\frac{1}{N}\sum_{t=1}^{N}\left|x_{t}-{\hat{x}}_{t}\right|$$ (11)

RMSE measures the difference between observed and predicted values, and it is computed using the following formula:

$$RMSE=\sqrt{\frac{1}{N}\sum_{t=1}^{N}{\left(x_{t}-{\hat{x}}_{t}\right)}^{2}}$$ (12)

MAPE represents the average of the absolute percentage errors in forecasts, where error shows the difference between actual and estimated values. This is defined by the formula provided below:

$$MAPE=\frac{1}{N}\sum_{t=1}^{N}\left|\frac{x_{t}-{\hat{x}}_{t}}{x_{t}}\right|*100$$ (13)

The best model will be chosen utilizing the RMSE, MAE, and MAPE criteria and forecasting will be made from 2022 to 2029for the best-selected model.

## 4. Results

The analysis starts with plotting the GDP graph spanning from 1969 to 2021. Moreover, we apply the candidate models to the data for comparison with the auto-regressive model, and necessary calculations are given in the interest of the reader. By plotting the series, we get a rare guess of whether the series is stationary or not. For checking stationarity statistically, we apply the ADF-test under the null hypothesis

H_o_ = Series has a trend and is not stationary

H_a_ = There is no trend or the series is stationary

**Fig 3:**
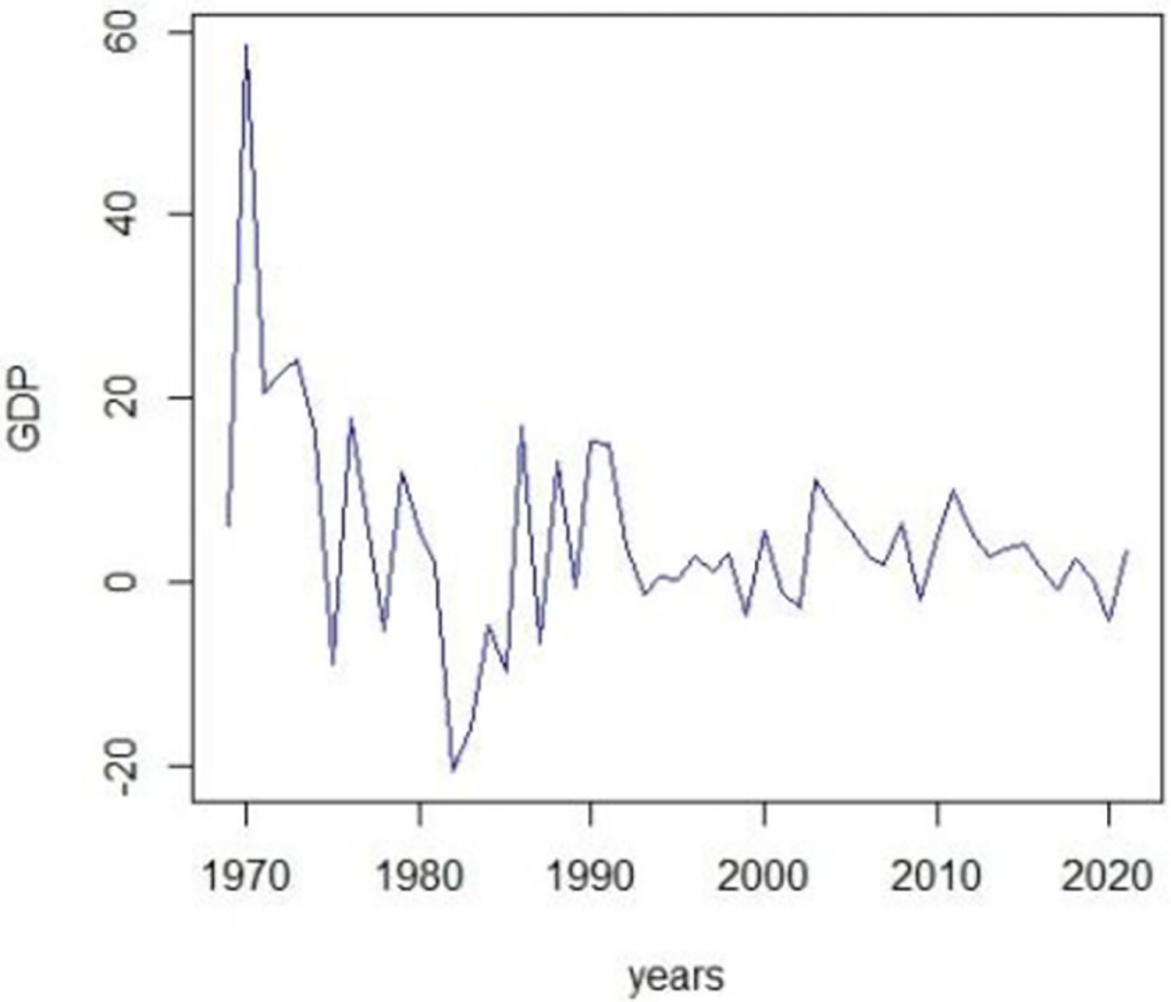
Time series plot.

**Fig 4:**
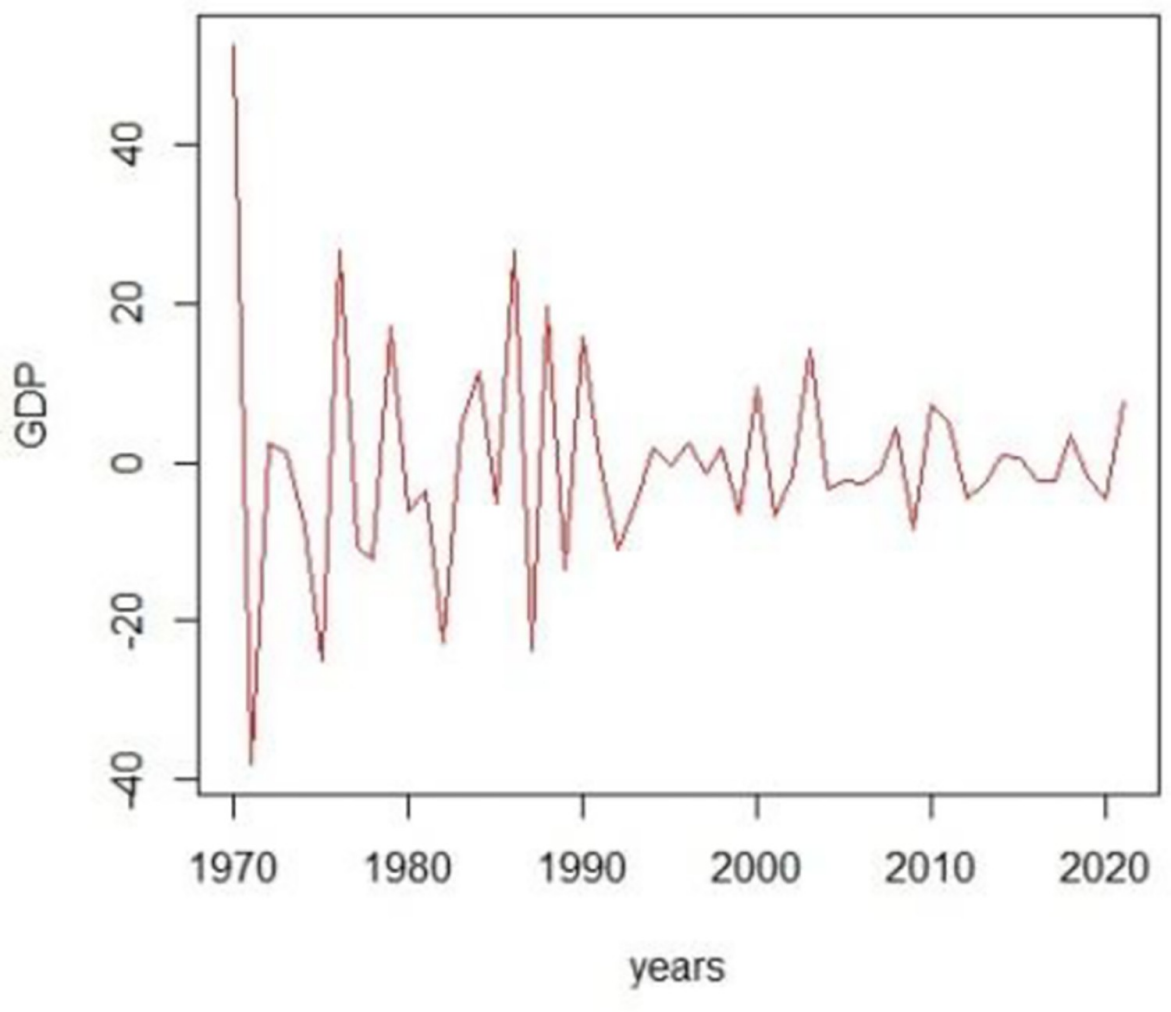
1^st^ difference time series plot.

**Fig 5:**
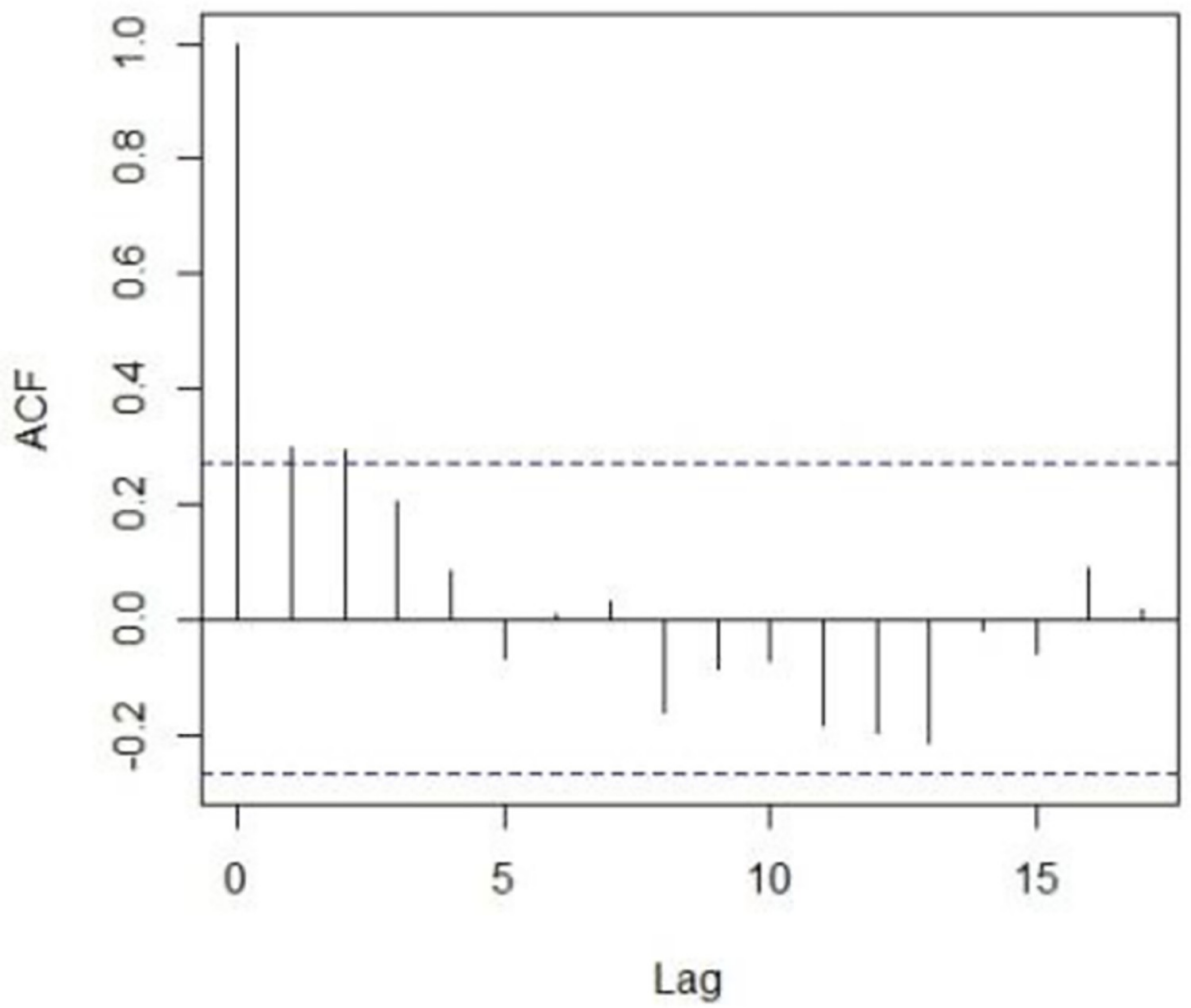
ACF of series GDP (%).

**Fig 6:**
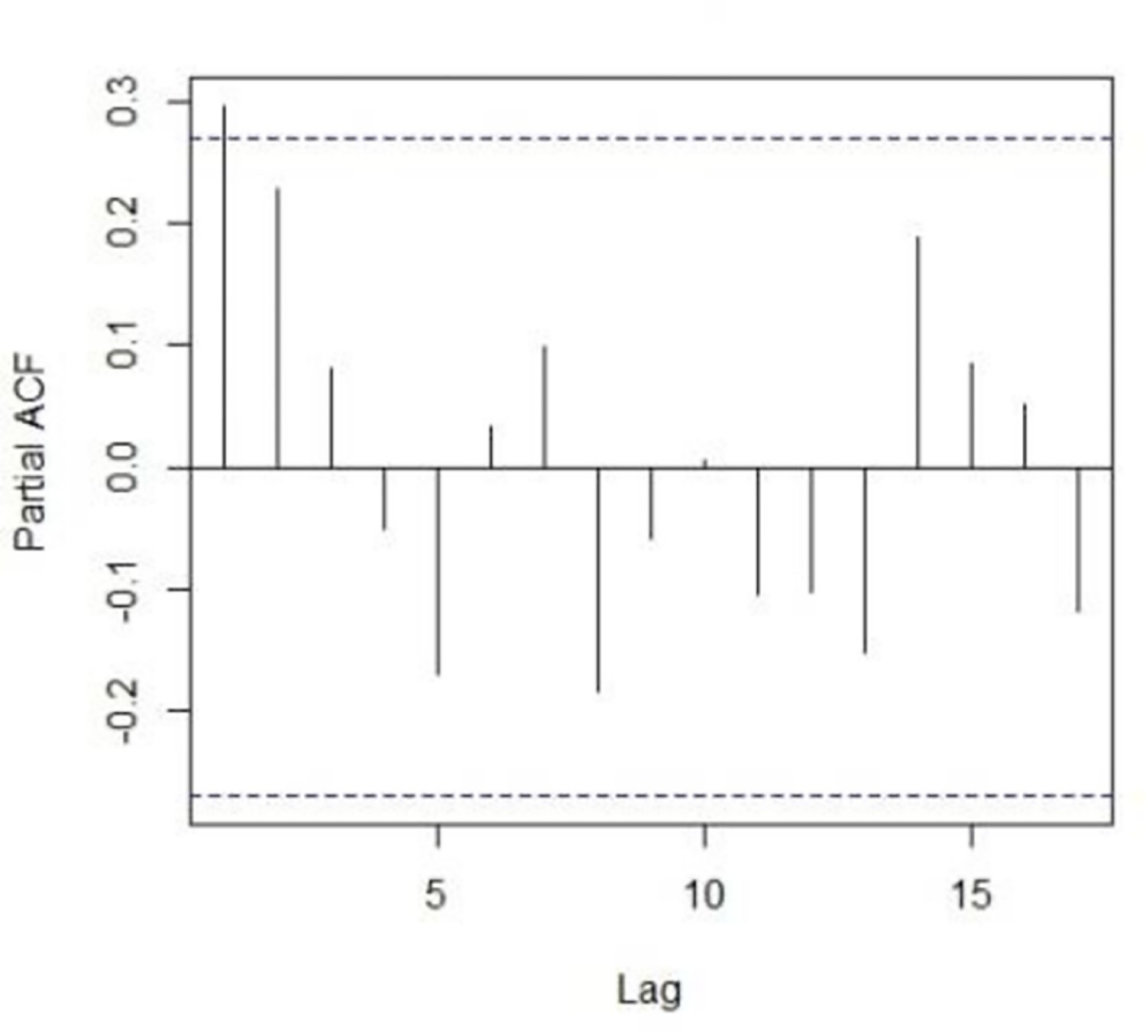
PACF of series GDP (%).

Based on the p-value we take decisions accordingly. If the series is stationary, we apply different combinations of ARIMA (***p*, *d*, *q***) candidate models. To make it stationary, we first take the series difference accordingly and make it stationary and then apply the ARIMA (***p*, *d*, *q***) candidate models where d is the order of differencing. The graph of the GDP and the ADF-test to check stationarity are given in Figs 3–6.

The first step of ARIMA modeling and its methodology begins with the identification of the model and it is achieved by checking the stationarity of the series. We proceed with the analysis by checking the stationarity of the GDP series. Augmented-Dicky fuller test will be applied to check the stationarity of the series. This test helps locate the non-stationarity component from the series. If the p-value is greater than the α = 0.05, the null hypothesis will be rejected and it will be concluded that the series is not stationary therefore we will make it stationary by applying the first difference transformation. From Fig 3, it can be examined that the series is not stationary. Non-stationarity can also be traced by plotting the correlogram of the series. Now to make the series stationary, we apply the differencing and then apply the Dicky-Fuller or ADF test to check the stationarity. after differencing the series and then applying the Dicky-Fuller test for stationary the results Dickey-Fuller = -15.6414, p-value = 0.000, with lag order = 3, shows that the series is stationary.

As the series is stationary now, the order of the candidate model can be rarely guessed by plotting the 1st difference correlogram of the series. There can be several combinations for the candidate model but we will select the model that has a lowest values of RMSE, MAE and MAPE. Among different candidates models chosen, the best-selected model is ARIMA (3,1,3) showing the lower values of all KPIs mentioned in Table 2.

**Table 2:** Candidate models for modeling GDP using ARIMA.

ARIMA(p,d,q)	RMSE	MAE	MAPE
**ARIMA (2,1,1)**	13.32	7.24	236.38
**ARIMA (3,1,4)**	12.73	7.42	248.39
**ARIMA (3,1,3)**	11.33	6.76	287.73

All these checks on residuals are executed using the R function checkresiduals(). This function generates a time plot, ACF plot, and a histogram of the residuals along with the normal curve. The null hypothesis of the Box Ljung box test states that the auto-correlation between the residual terms is zero or in other words, the model does not exhibit a lack of fit. The diagnostic checks of the residuals concluded that Q*-statistic = 7.8443 with the p-value = 0.097 concluding that the selected model ARIMA (3,1,3)does not exhibit a lack of fit and no-auto correlation exists between the residuals.

### 4.1 Hybrid model

The individual time series modeling methodologies i.e. ARIMA, TBATS, ETS, and NNAR were combinedby using the approach ofZhang [25] who developed a hybrid approach that combines and applies both the linear and nonlinear models in separate structures for modeling linear and nonlinear components of a time series. According to him, we have the following;

$$y_{t}=L_{t}+N_{t}$$ (14)

here, *yt* is the observation at the time y_t_ and *L*_*t*_, where *L*_*t*_ and *N*_*t*_ show the linear and non-linear parts of the series data, respectively. In the first step, linear time series i.e. ARIMA is fitted and modeled on the linear component, and the corresponding forecast ${\hat{L}}_{t}$ at the time *t* is obtained then the residuals from the linear model will contain only the nonlinear relationship. i.e.

$$e_{t}=y_{t}-{\hat{L}}_{t}$$ (15)

where ${\hat{L}}_{t}$ shows the forecasted value for time *t* from the linear time series model i.e ARIMA. By modeling the residuals using the non-linear models, the non-linear relationship can be explored. With the n inputs, the non-linear model for residuals will be;

$$e_{t}=f(e_{t-1},e_{t-2},\ldots ,e_{t-n})+\in_{t}$$ (16)

here *f* are the nonlinear functions determined by the non-linear models, Same approach was applied to combine the ARIMA with other nonlinear time series approaches. Denoting the forecast from Eq (16) as ${\hat{N}}_{t}$, the combined forecast can be expressed below;

$${\hat{y}}_{t}={\hat{L}}_{t}+{\hat{N}}_{t}$$

Specifically speaking the hybrid models are achieved in two steps. Initially,the ARIMA model is applied and used to analyze the linear pattern of the series, and in the second step, non-linear time series models NNAR are applied to modeled residuals from the ARIMA model. Since the ARIMA model can not analyze the non-linear pattern of the series, the linear pattern will contain information about the non-linear patterns. The results from the non-linear models will be used as the predictions of the error terms for the ARIMA model as well as the non-linear models. Thebest-selected of all models was evaluated by forecast accuracy measures of RMSE, MAPE, and MAE.

For the application of the NNAR model, we proceeded as follows;

(I) first, theBox–Cox transformation was applied before estimating the model.
(II) secondly, the optimum number of non-seasonal lags p was identified for AR (P) process then the P lag was set to 1 and the optimal number of neurons identified was estimated by the formula; $k=\frac{p+P+1}{2}$. Here p = 5 and P = 3, where p shows the embedding dimension for non-seasonal time series.

5 non-seasonal lags have been used as input nodes. In our model, there are 3 hidden layers. Results show that both neural network and hybrid models are much better in accuracy than the conventional linear and non-linear models. For longer time horizons, the NNAR model gives a comparable performance to the ARIMA, ETS, and TBATs models.

**Fig 7:**
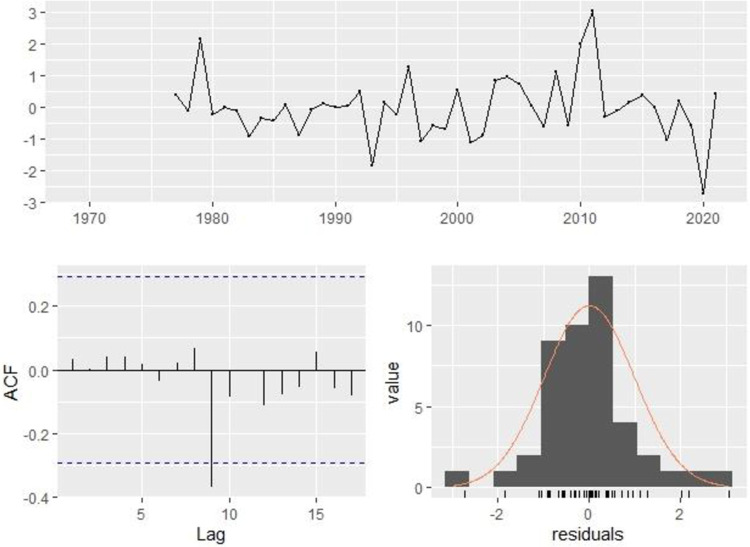
Diagnostics plots of residuals for NNAR (5,3).

Since the MAE values are lower than 10, all predictive models can be considered highly accurate. Similarly, the proposed forecasting approaches demonstrate significantly improved performance with lower mean absolute error. Among the hybrid models (ARIMA-NNAR, ARIMA-ETS, and ARIMA-TBATS) compared in Table 3, there is a consecutive decrease in values. Notably, ARIMA-NNAR exhibits the lowest values for RMSE (7.08), MAE (5.06), and MAPE (107.3) among the hybrid models. The identification of hybrid models was achieved using the "hybrid model()" function within the "forecasts hybrid" package in the R environment. There is a decline in the values of RMSE, MAE, and MAPE from ARIMA, ETS, TBATS to NNAR. The ARIMA model exhibits the highest KPI values, while ETS has a higher value compared to TBATS and NNAR. NNAR surpasses all individual time series models as well as hybrid models, showcasing RMSE (4.12), MAE (2.91), and MAPE (72.63). The hybrid model outperforms other hybrid models, while NNAR performs better among all linear and non-linear models. To validate the NNAR model, we proceed to the third step of the Box-Jenkins methodology, which involves diagnostic checking and applying normality tests to the residuals of the selected model. The Shapiro-Wilk normality test is employed to assess the normality of the model’s residuals. For the full sample data, the Shapiro-Wilk test yields a value of W = 0.98633 and a p-value of 0.2694, indicating that the null hypothesis is not rejected. Additionally, the Ljung-Box test statistics Q* = 11.776 with a p-value of 0.488 suggest that the model exhibits no lack of fit and the residuals are uncorrelated. In Fig 7, it can be observed that the NNAR (5,3) model incorporates all the available information. The residuals’ mean is close to zero, and no significant correlation is found within the residual series. The time plot of residuals shows a consistent variation across historical data, except for a single outlier, implying consistent residual variance. This is further supported by the histogram of residuals. However, it’s important to note that the histogram suggests a slight deviation from normality due to a left-tailed outlier, visible as a peak in the GDP at 58.647 in the 1970s.

**Table 3:** Comparison of different training individual and hybrid time series models using model selection criterions.

Training Model	RMSE	MAE	MAPE
**ARIMA**	11.33	6.76	287.73
**ETS**	10.71	6.84	126.25
**TBATS**	9.62	6.78	141.10
**NNAR**	4.12	2.91	72.63
**ARIMA-NNAR**	7.08	5.06	107.31
**ARIMA-TBATS**	9.57	6.21	109.32
**ARIMA-ETS**	7.12	5.07	126.91

**Fig 8:**
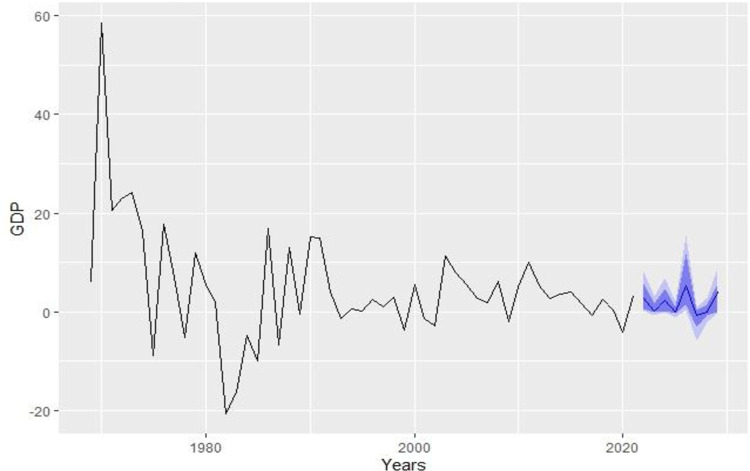
Forecast of KSA GDP(%) by NNAR (5,3).

Subsequently, the selected NNAR model has been utilized with sample data to predict forthcoming GDP values. When comparing hybrid and individual models, it becomes evident that NNAR (5,3) outperforms all other models in terms of MSE, RMSE, and MAPE. Furthermore, Fig 8 presents the forecasted values.

of GDP from 2022 to 2029. As indicated in Table 4, the NNAR model predicts an annual GDP growth rate of approximately 4.053% until 2029 for Saudi Arabia.

**Table 4:** Forecast valuesGDP(%) with 80 and 95% CI by NNAR (5,3).

Years	GDP Anuual Growth (%)	80% CI		95% CI*	
		Lower	Upper	Lower	Upper
**2023**	1.358	-4.66	6.49	-7.21	9.28
**2024**	1.257	-4.04	7.10	-6.80	10.44
**2025**	-0.963	-5.92	5.90	-9.19	9.25
**2026**	5.249	1.38	11.73	0.327	15.32
**2027**	-0.756	-3.12	0.33	-5.75	1.15
**2028**	-0.020	-0.65	1.37	-2.09	2.66
**2029**	4.053	0.02	5.78	-0.21	9.08

**Note:** 95% forecast interval gives an interval within which we expect *y*_*t*_ to lie with a specified probability. Assuming that the forecast errors are normally distributed, 95% prediction interval for the h-step forecast is; ${\hat{y}}_{T+h|T}\pm 1.96{\hat{\sigma }}_{h}$ where $\hat{\sigma }$ is the residual standard deviation.

## 4.2. Discussion

The journey of Saudi Arabia’s GDP (KSA) from 1969 to 2021 has been nothing short of remarkable, marked by significant fluctuations. These fluctuations posed a considerable challenge for conventional forecasting methods like the Autoregressive Integrated Moving Average (ARIMA). ARIMA excels in deciphering linear data patterns but often falls short when confronted with the complexity of non-linear dynamics. Realizing the importance of studying the non-linear patterns and increase the accuracy of our modeling, our study embarked on a more advanced approach. We chose to employ and compared both linear and non-linear models, alongside hybrid models that blend these two approaches. This allowed us to comprehensively model the GDP data by capturing both linear and non-linear patterns and finally NNAR model outperformed among all models and gave predictions of 2023 close to the IMF benchmark prediction. It’s worth noting that this study represents a pioneering effort, as no prior research has delved into modeling KSA’s GDP using non-linear models. This quantitative analysis provides policymakers with clear guidelines to oversee and sustain GDP growth in alignment with Saudi Arabia’s vision for fostering sustainable economic development. Moreover, it is essential for policymakers to prioritize the cultivation of a knowledge-based economy and the advancement of high-technology industries, as these sectors play crucial roles in propelling economic growth and facilitating the generation of wealth. In accordance with the objectives outlined in the 2030 vision, policymakers should craft policies that revolve around the principles of a knowledge-based economy, thus further amplifying Saudi Arabia’s growth trajectory in the near future.

## 5. Conclusion

The GDP growth rate of KSA faced Predicting and modeling GDP is crucial for policymakers and governments to formulate effective economic strategies and policies. GDP annual growth rate serves as a key financial indicator and can be used to forecast economic recessions and progressions. In the case of Saudi Arabia, the annual GDP growth rate has averaged 4.83% from 1960 to 2021, reaching its peak at 24.20% in 1973. This study focuses on forecasting the annual GDP growth rate (%) of Saudi Arabia using data from 1960 to 2021. The study demonstrated that NNAR (5,3) type of ANN model used in time series forecasting outperformed all other models in predicting the real GDP annual growth rate of KSA. Our study findings are consistent with studies [19–23] in which NNAR model outperformed its competing models. Assuming the IMF forecasts are the industry benchmarks, the forecasted GDP by NNAR model of year 2023 (1.3) is close to the projection of IMF for 2023 which is 1.9(https://www.imf.org/en/Countries/SAU). This experimental study offers significant insights that can assist economic experts in formulating strategies to enhance GDP growth on an ongoing basis.

## 6. Limitations and future research

While our study has made significant strides in improving GDP growth rate predictions for Saudi Arabia, there are still some limitations worth considering. Firstly, our research is based on historical data up to 2021, and economic conditions can change rapidly. Therefore, the accuracy of our predictions for future years, such as 2023, may be influenced by unforeseen events or shifts in the global economic landscape and development.

For future research, it would be valuable to keep analyzing the latest data to improve and validate our predictive models continuously. We should also explore ways to make neural network models easier to understand and gain a deeper insight into the specific economic factors affecting Saudi Arabia’s GDP growth. Different configurations of neural networks can be constructed and results can be tested for each configuration. Moreover, it’s important to investigate how external factors like international trade dynamics, geopolitical events, and environmental factors impact Saudi Arabia’s GDP growth. This broader approach can offer a more complete understanding of the country’s economic path, aiding in making more informed policy decisions.
